# Telemedicine medical abortion at home under 12 weeks’ gestation: a prospective observational cohort study during the COVID-19 pandemic

**DOI:** 10.1136/bmjsrh-2020-200976

**Published:** 2021-02-04

**Authors:** John Joseph Reynolds-Wright, Anne Johnstone, Karen McCabe, Emily Evans, Sharon Cameron

**Affiliations:** 1 Queen's Medical Research Institute, University of Edinburgh MRC Centre for Reproductive Health, Edinburgh, UK; 2 NHS Lothian, Chalmers Centre, Edinburgh, UK; 3 Edinburgh Clinical Research Facility, Edinburgh, UK

**Keywords:** abortion, therapeutic, abortion, induced, COVID-19, health Services Research, mifepristone, reproductive health

## Abstract

**Background:**

In response to the COVID-19 pandemic, legislation and guidance were introduced in Scotland permitting medical abortion at home by telemedicine for pregnancies at less than 12 weeks’ gestation. Women had a telephone consultation with a clinician. Routine ultrasound was not performed. Medications and a low-sensitivity pregnancy test to confirm success of treatment were collected by or delivered to the woman, with telephone support provided as needed.

**Methods:**

A prospective cohort study of 663 women choosing medical abortion at home via the NHS Lothian telemedicine abortion service between 1 April and 9 July 2020. Interviewer-administered questionnaires were completed 4 and 14 days following treatment. Regional hospital databases were reviewed to verify abortion outcomes and complications within 6 weeks. Outcome measures included efficacy, complications and acceptability.

**Results:**

Almost all (642/663, 98.2%) the women were under 10 weeks’ gestation. For 522/663 (78.7%) women, gestation was determined using last menstrual period alone. Some 650/663 (98%) women had a complete abortion, 5 (0.8%) an ongoing pregnancy and 4 (0.6%) an incomplete abortion. No one was treated inadvertently beyond 12 weeks’ gestation, but one woman was never pregnant. One woman who had a pre-abortion ultrasound was later managed as a caesarean scar ectopic. There were two cases of haemorrhage and no severe infections. Some 123 (18.5%) women sought advice by telephone for a concern related to the abortion and 56 (8.4%) then attended a clinic for review. Most (628, 95%) women rated their care as very or somewhat acceptable.

**Conclusions:**

This model of telemedicine abortion without routine ultrasound is safe, and has high efficacy and high acceptability among women.

**Key messages**

- Telemedicine medical abortion at home in the first trimester without routine ultrasound is effective, with low complication rates and high acceptability.
- Telemedicine service models need flexibility and resources to accommodate those women requiring clinical review post-abortion including ultrasound assessment.
- If given the choice, most women would choose a telemedicine consultation again rather than an in-person visit.

## Introduction

Until recently, medical abortion care in Britain typically involved an in-person clinical consultation and a routine ultrasound scan to assess gestational age.^1 2^ Women had to take mifepristone in a clinical setting but for those less than 10 weeks’ gestation, there was the option to self-administer the second part of the treatment, misoprostol, at home. Women over 10 weeks’ gestation were admitted to a clinical facility for misoprostol.^3^

In March 2020, the COVID-19 outbreak in the UK resulted in legislation being introduced permitting home use of mifepristone.^4^ Recommendations from the Royal College of Obstetricians and Gynaecologists for the provision of abortion care were introduced that supported telemedicine consultations (telephone or video call), gestational assessment based on the date of last menstrual period (LMP) and delivery of medical abortion drugs to women eligible for this care at home.^5^ In Scotland, additional clinical guidelines were introduced that supported telemedicine provision of medical abortion at home up to 11 weeks and 6 days’ gestation.^4^

NHS Lothian is the sole provider of abortion care in Edinburgh and the surrounding region. Just over 2600 women receive abortion care annually.^6^ All abortion care including post-abortion contraception is provided at no cost to the patient, as is the norm in the National Health Service (NHS). As a result of the new legislation and clinical guidance,^4 5^ the service moved wholly to provision of abortion care by telemedicine and without routine ultrasound on 1 April 2020.

Globally, telemedicine provision of medical abortion has largely been carried out in areas where abortion access is restricted, but existing evidence suggests that rates of complete abortion, emergency admissions to hospital and serious complication rates are similar to those after abortion care in a clinical setting and that acceptability for women is high.^7–11^

We initiated an evaluation of the NHS Lothian telemedicine medical abortion service, based at the Chalmers Sexual and Reproductive Health Centre in Edinburgh. The aims were to determine the number of women having medical abortion at home without routine ultrasound, the efficacy of the procedure when delivered by this model, safety based on serious complications after treatment, and women’s acceptability of their care.

## Methods

### The telemedicine model

Anyone aged 16 years or older who contacted the service to discuss an unwanted pregnancy was given an appointment for a telephone consultation with a clinician.^2^ They were advised to visit the service website for more information on what to expect at the teleconsultation, including audiovisual resources.^12^ Those aged less than 16 years were routinely offered an in-person appointment.

During the teleconsultation, women were assessed for the need for ultrasound (LMP uncertain or >12 weeks ago, pain, bleeding, or significant risk factors for ectopic pregnancy).^4 5^ For eligible women (LMP <12 weeks ago, no contraindications to mifepristone and misoprostol) requesting medical abortion at home, informed consent was taken verbally, and arrangements made for collection of a medication pack from the service or for couriered delivery to the woman’s home. In line with national guidance introduced in relation to COVID-19, anti-D prophylaxis was not provided or considered necessary for rhesus-negative women having medical abortion in the first trimester.^4 5^ The contents of the medication pack included prophylactic antibiotics and are shown in online supplemental table 1. Post-abortion contraception was discussed during the consultation, and condoms and short-acting hormonal contraception were included in the medication pack if women wished.^13^ Those who requested long-acting reversible contraception (LARC) were offered a clinic appointment 2 weeks later.

10.1136/bmjsrh-2020-200976.supp1 Supplementary data

For women requiring an in-person consultation and/or ultrasound, this pack was provided at that visit. For women over 12 weeks’ gestation or choosing surgical abortion, the existing arrangements for direct admission to the local hospital for this procedure were made.^14^ An overview of the telemedicine care pathway can be seen in online supplemental figure 1.

### Evaluation

We conducted a prospective observational study of women having medical abortion at home via telemedicine from its instigation on 1 April 2020 until 9 July 2020. We prospectively collected data on the numbers requiring a pre-abortion ultrasound, as well as routinely collected data on gestation, demographic characteristics, reproductive history and choice of post-abortion contraception. For the purposes of the evaluation only, women were contacted by telephone by a researcher for an interviewer-administered questionnaire on days 4 and 14 after misoprostol administration.

The day 4 questionnaire asked about acceptability of the care and preparedness for the procedure. The day 14 questionnaire recorded the result of the self-performed low-sensitivity urine pregnancy test (LSPT) to confirm the success of the procedure,^15^ method of contraception provided and women’s ratings on the importance of different parts of the telemedicine model for future service development. Responses were either binary (yes/no), rated on a five-point Likert scale (eg, very acceptable to very unacceptable) or by selection from a predefined list of possible options (online supplemental figure 2).

If research staff received a clinical query or elicited information about a possible complication, women were transferred to the abortion service clinical advice line for assessment and care as needed. All these calls for advice were recorded in patient electronic records.

The outcome of the pregnancy (complete abortion, incomplete abortion, ongoing pregnancy) and complications (haemorrhage defined as ≥500 mL blood loss, severe infection defined as requiring intravenous antibiotics) were verified at 6 weeks after abortion through a review of both the regional hospital electronic patient records (TRAK) and the sexual health service records (NaSH).

The primary outcome of the study was efficacy of medical abortion, with success of abortion defined according to the Medical Abortion Reporting of Efficacy (MARE) guidelines as successful expulsion of pregnancy without the need for surgical intervention.^16^ Secondary outcomes were severe complications (haemorrhage and severe infection), adverse outcomes such as undiagnosed ectopic pregnancy, gestation beyond 12 weeks, unscheduled contact with the service, acceptability of the telemedicine service, and contraception uptake.

### Statistics

A descriptive analysis has been presented: continuous data as mean and standard deviation (for example, age) and categorical data as numbers and percentages of total responders. In some instances due to small numbers categorical responses may have been grouped; however, unless otherwise indicated we have treated all responses in the categories they were collected in. All statistical analysis was performed using SAS Enterprise Guide v 7.15 (SAS Institute Inc., Cary, NC, USA) and Microsoft Excel 2016 by an independent statistician.

### Approvals

The project received approval from the NHS Lothian Sexual and Reproductive Health Service Quality Improvement Team and was not deemed to require ethical approval following review by the local NHS Research Ethics Committee scientific officer.

### Patient and public involvement

Patients and members of the public were not directly involved in the design of this study.

## Results

During the study period, 826 women had a teleconsultation. Thirty-five (4.3%) women chose to continue with the pregnancy, 31 (3.8%) had a miscarriage diagnosed so did not proceed to abortion and the outcome was not known in 2 (0.2%) cases. Of the remaining 758 women who proceeded to abortion, 663 (87%) had a medical abortion at home and were included in the study cohort. The remainder had either a medical or surgical abortion in a hospital setting or were referred to an external provider in England for abortion as they were over 20 weeks’ gestation and so the abortion care could not be provided at the local hospital.

### Characteristics of women

Table 1 summarises the demographic information and gestational age data. The mean (SD) age was 27.6 (6.6) years (range 16 to 50 years). In 522/663 (78.7%) cases the gestation was determined using LMP alone. In 141 (21.3%) cases a pre-abortion ultrasound was performed for uncertain gestation (n=95; 14.3%) or to confirm that pregnancy was intrauterine (n=33; 5.0%). Thirteen (2%) women had already had an ultrasound at a different service before attending. Capturing gestation in weeks, 56% of women had a gestation of 5 weeks to 6 weeks and 6 days.

**Table 1:** Demographic characteristics of the telemedicine cohort.

Characteristic	Patients who had medical abortion at home	
	n	%
Total	663	100
SIMD*		
Unclassified	2	0.3
1	120	18.1
2	198	29.9
3	101	15.2
4	111	16.7
5	131	19.8
Reproductive history		
Previous live birth	324	48.9
Previous medical abortion	222	33.5
Previous surgical abortion	39	5.9
Previous miscarriage	125	18.9
Previous ectopic pregnancy	12	1.8
Smoking status		
Never	367	55.4
Past	60	9
Current	193	29.1
Unknown	43	6.5
Gestation (weeks+days)**†**		
<3+6	1	0.2
4–4+6	72	10.9
5–5+6	188	28.4
6–6+6	182	27.5
7–7+6	112	16.9
8–8+6	58	8.7
9–9+6	29	4.4
10–10+6	15	2.3
11–11+6	6	0.9

*Scottish Index of Multiple Deprivation.^24^

†For 141 patients the gestational age was taken from an ultrasound result. For all the remaining patients the gestational age was taken from the last menstrual period (LMP).

### Outcome of medical abortion

The outcomes of medical abortion are shown in table 2. A complete abortion took place in 650/663 (98%) cases. Eight of the nine failed abortions (ongoing or incomplete) were at gestations <10 weeks. No one was known to have been treated inadvertently beyond 12 weeks’ gestation.

**Table 2:** .Efficacy of medical abortion

Outcome	Cohort (n=663)* n (%)
Complete abortion	650 (98.0)
Ongoing pregnancy	5 (0.8)
Incomplete abortion	4 (0.6)

*The remaining four outcomes were: n=2 (0.3%) did not proceed to take the abortion medication and self-referred for antenatal care to continue the pregnancy. n=1 was presumed to have a caesarean scar ectopic pregnancy. She had an ultrasound at the hospital early pregnancy unit before referral for abortion that reported a small sac low within the uterus with a possible fetal pole. She re-presented 1 week after medical abortion with persisting pregnancy symptoms. A further ultrasound had unchanged findings and given her history of caesarean delivery, it was considered as a likely caesarean scar ectopic. She was managed by the hospital and treated uneventfully with methotrexate. n=1 reported no bleeding after medical abortion. She had an empty uterus on ultrasound 4 days following misoprostol and a negative serum human chorionic gonadotrophin (hCG), suggesting that she was probably never pregnant.

### Complications and unscheduled care after abortion

Some 16/663 (2.4%) women made unscheduled attendances to the hospital. Two (0.3%) of these women were admitted with haemorrhage but neither required transfusion (both <10 weeks’ gestation). A further 13 (2%) women attended hospital with pain and/or bleeding but required only observation (n=7) or intravenous fluids (n=3) or were sent home with oral antibiotics (n=3). One woman was admitted with an unrelated medical event. No one presented with severe infection requiring intravenous antibiotics.

Some 123/663 (18.5%) women telephoned the abortion service for clinical advice. In 67 (10.1%) cases only telephone advice was required. In 56 (8.4%) cases a clinic visit was subsequently scheduled because of a positive or invalid LSPT (n=34), symptoms of continuing pregnancy (n=18) or persistent pain (n=4). In total, a post-abortion ultrasound was conducted in 66 (9.9%) cases (hospital admissions and clinic visits combined).

### Contraception

The method of contraception provided to women is shown in table 3. The the most common method was the progestogen-only pill (desogestrel 75 µg) provided to 423 (63.8%) women. Fifty-eight (8.7%) women received long-acting reversible contraception (LARC).

**Table 3:** Contraceptive method supplied (based on day 14 questionnaire response and clinic records)

Contraceptive method	Cohort (n=663) n	%
POP	423	63.8
COCP	49	7.4
Condoms	62	9.4
Injectable*	19	2.9
Implant*	17	2.6
IUD*	19	2.9
IUS*	20	3
No method	40	6
Patch	14	2.1

*These methods were initiated if women attended clinic for a pre-abortion ultrasound appointment (injectable and implant only) or at a rapid access clinic at a later date.

COCP, combined oral contraceptive pill; IUD, intrauterine device; IUS, intrauterine system; POP, progestogen-only pill.

### Acceptability

Almost all (n=652, 98.3%) the women provided responses to at least one follow-up questionnaire. Complete questionnaires at both day 4 and day 14 post-abortion were available for 605 (91.3%) women. Forty-five (6.8%) women responded to the day 4 contact only and 2 (0.3%) only responded to the day 14 contact.

Some 627 (94.6%) women stated at day 4 that they were ‘very’ (n=516, 77.8%) or ‘somewhat prepared’ (n=111, 16.7%) for their treatment following the telemedicine consultation.

Similarly, 628 (94.7%) women rated the abortion experience as ‘somewhat’ (n=43, 6.5%) or ‘very’ (n=585, 88.2%) acceptable at day 4. Asked on day 14, 588 (88.7%) stated that they would opt to have treatment at home again if they needed another abortion.

Regarding acceptability of the remote consultation (day 14), 574 (86.6%) women rated it as ‘somewhat’ (n=24, 3.6%) or ‘very’ (n=550, 83%) acceptable. Some 473 (71.3%) women stated that they would opt for a telephone consultation again if they required an abortion in the future.

#### Features of importance in an ideal abortion care service

On day 14, women were asked to rate the importance of the features listed in table 4 for a ‘perfect abortion service’. The feature rated by most respondents as ‘important’ or ‘very important’ was ‘being able to collect all my medications from a pharmacy’ by 366/567 (64.6%) women. Ultrasound was rated ‘unimportant or very unimportant’ by 302/569 (53.1%) women.

**Table 4:** Aspects of a perfect abortion service from the day 14 questionnaire (ie, how important each aspect was considered by women)

Aspect	Unimportant		Neutral		Important	
	n	%	n	%	n	%
Having an ultrasound scan	302	53.1	116	20.4	151	26.5
Evening face-to-face clinic	133	23.8	206	36.2	230	40.4
Evening telephone consultation	61	10.8	208	36.7	298	52.6
Skype or video consultation	383	67.9	78	13.8	103	18.3
A mobile phone app to send or receive information in advance	92	17.5	101	18.0	367	65.5
Online booking	66	12.1	119	21.8	361	66.1
Medication posted to me	74	13.0	152	26.8	342	60.2
Medication that could be collected from a local pharmacy	58	10.2	143	25.2	366	64.6
Able to get the treatment from my general practitioner	112	19.8	160	28.2	294	51.8

Some women did not answer certain questions but responded to others and so the denominators and totals may vary slightly between variables.

## Discussion

Telemedicine medical abortion at home was used by almost nine out of ten women and, of these, only two out of ten women required a pre-abortion ultrasound. The study showed high rates of complete abortion, low rates of complications and low rates of unscheduled contact. These findings are similar to previous cohort studies from this service of women who took misoprostol at home having had routine ultrasound and mifepristone in clinic.^14 15 17^ Women reported high levels of preparedness to use the medications in their own homes. Indeed, acceptability of care was high, and the majority of women expressed a preference for choosing this model of care again in the future.

This study provides support for continuing this model of care beyond the current pandemic. There was one instance of unnecessary treatment, where the woman had probably never been pregnant. The only ectopic pregnancy in the cohort had a pre-abortion ultrasound and was initially thought to have an intrauterine pregnancy. This highlights how routine ultrasound can provide false reassurance of intrauterine pregnancy.^18^ Indeed, the treatment failure and persistence of pregnancy symptoms may have expedited diagnosis and treatment of this ectopic.

Not requiring pre-abortion ultrasound potentially expands the range of healthcare providers who can offer medical abortion,^19^ as recommended by the World Health Organization (WHO).^20^ There is also existing evidence to support the safety of medical abortion provision without ultrasound based on LMP alone.^21^ When asked, more than half of the women in our study considered the use of routine ultrasound to be ‘unimportant’ and over half of the women also considered that general practitioners as abortion providers would be an important future development.

In our study, rates of telephone contact to the service for a concern related to the abortion were similar to our previous studies.^14 15^ However, we did observe a higher proportion of women requiring a post-abortion clinic attendance (8.4%) compared with our former service model with routine pre-abortion ultrasound and clinic-administered mifepristone with misoprostol at home (2.7%).^14^ The majority of the post-abortion clinic visits were to exclude continuing pregnancy following a positive or invalid LSPT. It is possible that the telephone contact from the researcher triggered more of these post-abortion visits since they actively questioned women about the pregnancy test result, rather than leaving them to call the service if they had a concern. It is also likely that without the knowledge of a pre-abortion ultrasound, staff have a lower threshold for arranging a clinic review post-abortion to minimise the chance of missing an ectopic or a pregnancy that may have been at a more advanced gestation. This need for post-abortion clinical support needs to be factored into provision of telemedicine abortion.

Most women received contraception, but this was mostly oral contraceptive pills, which are less effective than LARC methods^22^ but can be initiated immediately if a woman has all her care by telemedicine. Fewer than one in ten women in this study received LARC in contrast to previous studies at our service where around one-third of women received LARC.^14^ Although, strategies such as ‘fast track’ to post-abortion contraception clinics can facilitate access to LARC,^23^ it is possible that the pandemic may have played a role in women’s choice both to use contraception and also the method. Some women may have opted not to use LARC as it required a further visit to a clinic with risk of exposure to the virus, plus the convenience of receiving oral contraception in the treatment pack.

Our study also showed that there is support among women for several simple modifications to the service that could be developed in the future, including expanding the range of options for accessing medications such as postal delivery of medical abortion drugs or collection from a local pharmacy.

This study had an extremely high rate of follow-up for all outcomes. This may be in part because women were at home during the COVID-19 lockdown restrictions and so were easily contactable. Using the electronic patient record for the entire region, we were able to actively search for all serious complications within a 6-week timeframe, and verify abortion outcomes rather than relying on self-reporting at an earlier timepoint alone. Nevertheless, the study size, while considerable, is still too small to detect changes in rare events. While possible, it is extremely unlikely during this period when the study was conducted that many women will have travelled out of the region and suffered a complication. Of course, the telemedicine service was devised and evaluated during the COVID-19 pandemic and so is not yet embedded under ‘normal’ circumstances, where the proportion of women choosing or being invited for in-person care may be different. In addition, qualitative research is required to provide more nuanced information on women’s views on the acceptability of the telemedicine medical abortion service.

## Conclusions

This model of telemedicine medical abortion without routine ultrasound is safe, and has high efficacy and high acceptability among women. This study provides support for continuation of this model of care in this setting beyond the current pandemic.
