# Supplementary material for: Telemedicine medical abortion at home under 12 weeks’ gestation: a prospective observational cohort study during the COVID-19 pandemic
Source: BMJ Sex Reprod Health. 2021 Feb 4;47(4):246–51. doi: 10.1136/bmjsrh-2020-200976 (PMC7868129; doi:10.1136/bmjsrh-2020-200976)
Supplement: Supplementary data [file bmjsrh-2020-200976supp001.pdf]

Supplementary Figure 1: Patient journey through telemedicine early medical abortion care (CCBY4.0 John Reynolds-Wright available from https://flic.kr/p/2j8HtKK)

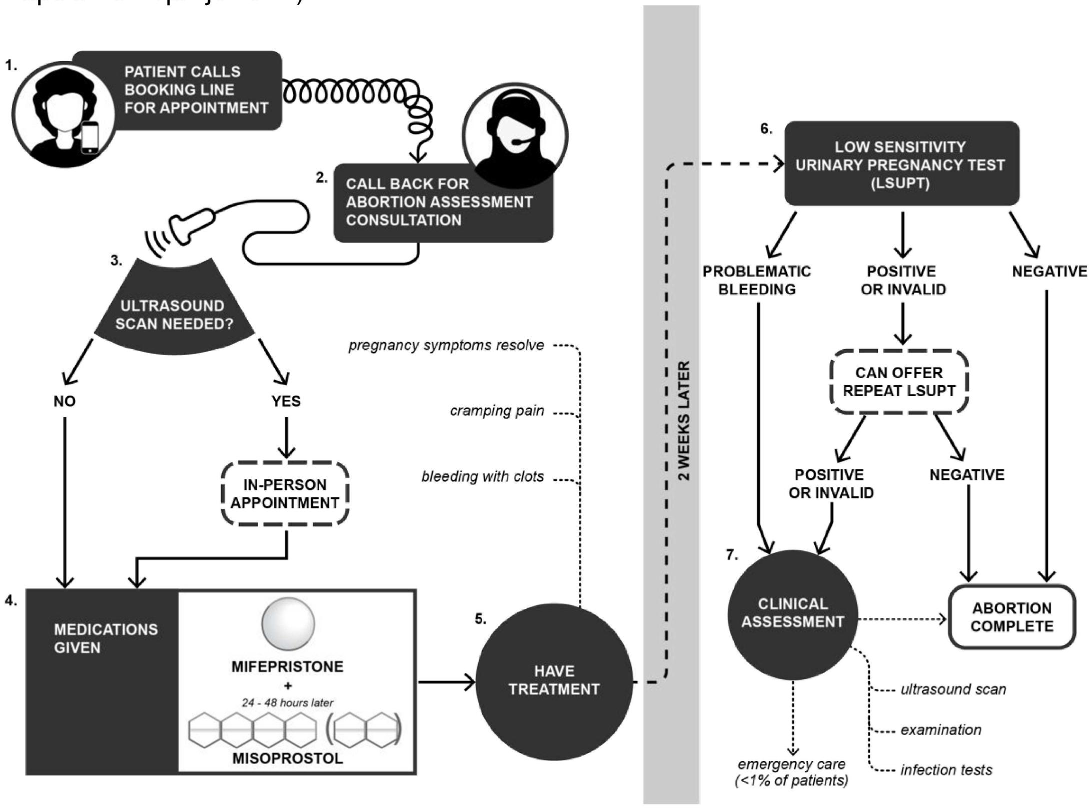

Supplementary Figure 2: Questionnaires

DAY FOUR QUESTIONS

Question 1:

Did you take the first tablet (mifepristone, 1 tablet that you swallow)?

Circle: Yes / No

If yes, do you remember when:

Date:________________________________ Time:__________________________

Or circle: Don't remember

Question 2:

Do you remember the date and time you took the first dose of your misoprostol tablets (4 tablets under the tongue or inside the vagina)?

Circle: Yes / No

If yes:

Date:________________________________ Time:__________________________

Did you take the tablets (circle): under the tongue / inside the vagina / between your cheek and gum ?

Question 3:

Did you use any additional doses of misoprostol? (2 more tablets under the tongue or inside the vagina)

Circle: Yes / No

If yes:

How many further doses (i.e. pairs of tablets) did you take? ___________________

Question 4:

Do you remember the date and time that you passed the pregnancy?

Circle: Yes / No / Unsure

If yes:

Date:________________________________ Time:__________________________

Question 5:

How acceptable did you find having your abortion treatment in your own home? (please circle)

- Very acceptable
- Somewhat acceptable
- Neutral
- Somewhat unacceptable
- Very unacceptable

Question 6:

How prepared did you feel for having your abortion treatment at home? (please circle)

- Very prepared
- Somewhat prepared
- Neutral
- Somewhat unprepared
- Very unprepared

Question 7:

If you had to have this treatment again, where would you rather have it:

- At my own home
- In hospital
- Have a surgical procedure
- Not sure

DAY FOURTEEN QUESTIONS

Question 8: When did you do your pregnancy test and what was the result?

LoSUPT date: ____

LoSUPT result: Positive / Negative / Invalid

Question 9: Were you given a supply of contraceptive pills? Yes / No

If Yes, have you started them: Yes / Not yet but I will /
No, I am going to use something else

Question 10:

Looking back, how acceptable did you find having your consultation remotely?

- Very acceptable
- Somewhat acceptable
- Neutral
- Somewhat unacceptable
- Very unacceptable

Question 11:

Looking back, if you were able to choose a type of consultation for this procedure which would you choose?

- Face-to-Face
- Telephone
- Video call
- Unsure

Question 12:

If you were to design the perfect service, which of the following would be important to you:

	Very Unimportant	Somewhat Unimportant	Neutral	Somewhat Important	Very Important
Having an ultrasound scan					
Evening face-to-face clinic					
Evening telephone consultation					
Skype or video consultation					
A mobile phone app to send/receive information in advance					
Online booking					
Medication posted to me					
Medication that could collect from a local pharmacy					
Able to get the treatment from my GP					

Supplementary Table 1. Medication pack for medical abortion at home.
*FSRH = Faculty of Sexual and Reproductive Healthcare UK.

Abortion medications	Mifepristone 200mg Oral<br><br>24-48 hours later<br><br>Misoprostol 800micrograms sublingual/vaginal/buccal (per patient preference)<br><br>Plus a further 1 x 400micrograms of misoprostol if no bleeding within 4 hours of first dose<br><br>For 10-11+6 weeks only:<br>a further 400micrograms of misoprostol if not passed pregnancy by 4 hours of last dose<br><br><10 weeks provided 1200 mcg misoprostol total and 10-11+6 weeks provided 1600 mcg misoprostol total
Analgesia	Dihydrocodeine 30mg<br>Women advised to purchase their own supply of paracetamol and ibuprofen.
Antibiotics	Doxycycline 100mg twice daily for 7 days
Antiemetic (if required)	Cyclizine 50mg oral
Confirmation of abortion	Low Sensitivity Urine Pregnancy Test 1000iu at 14 days
Contraception	Pills, patches, rings and condoms supplied in pack.<br>Combined hormonal methods issued in accordance with FSRH* advice.<br>Long acting reversible contraceptives provided at rapid access clinic and bridging method offered.
Information	Detailed step-by-step information provided as written leaflet included in pack.
